# In Vitro Comparison of Raypex 6 and Endopilot Using a Novel, Computer-Aided Measurement System, for Determining the Working Length

**DOI:** 10.1371/journal.pone.0134383

**Published:** 2015-08-03

**Authors:** David Christofzik, Falk Schwendicke, Christian Flörke, Alexander Härtl, Christof Dörfer, Birte Größner-Schreiber

**Affiliations:** 1 Clinic for Conservative Dentistry and Periodontology, School for Dental Medicine, Christian-Albrechts-University Kiel, Kiel, Germany; 2 Department of Operative and Preventive Dentistry, Charité Centrum 3, Charité-Universitätsmedizin Berlin, Berlin, Germany; 3 Department of Computer Science, University of Miami, Coral Gables, Florida, United States of America; The Forsyth Institute, UNITED STATES

## Abstract

**Introduction:**

Experimental evaluation of endometric devices usually relies on visual, subjective detection of the apical constriction to determine the accuracy of measurements. The aim of the present study was to analyze the accuracy of measurements of Raypex 6 and EndoPilot using a novel, objective image-analysis system.

**Methods:**

Onehundred and twenty teeth were randomized and allocated to three groups: After coronal flaring, either Raypex 6 or EndoPilot were used to determine the endodontic working length during instrumentation using manual files (RPM and EPM group respectively). In addition, EndoPilot was used for continuous, automatic measurement during rotating instrumentation (EPA group). If the working length had been reached according to endometric results, the files were fixed in place. Tooth and file were then embedded and prepared for analysis. Subsequently, the distance between the tip of the file and the apical constriction (D_AC_) or the apical foramen (D_AF_) was calculated using trigonometric analysis and the position of the file relative to AC and AF was analyzed.

**Results:**

Both inter- and intra-examiner-reliability of the trigonometric analysis were nearly perfect (ICC = 0.999, p<0.001). D_AC_ was not significantly different between groups (p>0.05, t-test). D_AF_ was significantly decreased when EPA had been used compared to EPM (p<0.05, Exact-test). EPA resulted in files being positioned beyond AF significantly more often than the other two methods (p<0.01).

**Conclusions:**

All methods allowed reliable detection of AC. However, EPA significantly increased the risk of overpreparation. Objective, digital assessment based on image analysis was suitable to compare the accuracy of different endometric devices.

## Introduction

The long-term success of endodontically treated teeth is determined by several factors like the presence, degree and extension of periapical inflammation or infection, a sufficient root filling as well as an adequate coronal seal [1, 2]. Longer average retention time of teeth increase endodontic treatment needs, and with rising life expectancy the number of performed root canal treatments per patient increases as well [3]. One of the most important factors for the long-term prognosis of a root filled tooth is the length of instrumentation and the position of the apical seal [4–7]. If the instrumentation remains shorter than desired (under preparation) in the case of necrotic pulp, bacteria remain and survive apically to the instrumented areas [8, 9]. Over preparation transports bacteria and infected tissue into the periapex, thereby compromising healing and regeneration of periapical tissues [10].

Radiographic measurement allows detection only the radiological apex [11], which is commonly found 1 mm beyond the apical constriction, with great variations from this rule [12, 13]. Thus, a solely radiographical determination of the working length is associated with a high risk of overpreparation [14]. In contrast, endometric measurements were shown to be a reliable, accurate method to estimate the length of the root canal [4, 15] and to determine the interval between the anatomical apex and the apical constriction [4, 16].

A major problem when experimentally assessing the accuracy of different endometric measurement systems is the identification of the apical constriction (AC): Often, a visual assessment using magnification aids was performed, which is prone to subjectivity and of limited reliability [4, 17, 18]. The present study introduces a newly designed image analysis software system, which allows reliable and objective identification of the AC. The system is used to compare two new endometric measurement devices, which use different measurement techniques. Both Raypex 6 (VDW, Munich, Germany) and EndoPilot (Schlumbohm, Brokstedt, Germany) allow manual assessment of the endodontic working length. In addition, EndoPilot allows continuous measurements during rotating instrumentation. Raypex 6 succeeds Raypex 5, which was shown to determine the working length with high reliability [17], whereas the EndoPilot uses a new detection method based on an impulse measuring system, thereby allowing increased measurement frequency and thus improving the accuracy for continuous measurements [19, 20]. The aim of this study was to compare the accuracy of manual and continuous endometric measurements in the assessment of the endodontic working length using an objective digital analysis system.

## Materials and Methods

### Specimen preparation

120 sound, caries-free, unrestored permanent teeth with complete root formation, which were extracted due to surgical or periodontal concerns with informed consent and under a protocol approved by the school’s IRB (Ethic committee of the Christian-Albrechts-University Kiel D444/10), were selected. Written consent was given, and only adult teeth were used. Teeth were cleaned and those with apical resorptions or vertical fractures excluded; specimens were stored in 0.08% thymol solution. Molars (n = 60) and single-rooted teeth (premolars and anteriors n = 60) were block-randomized and allocated to three experimental groups (Raypex, discrete measurement [RPM], EndoPilot manual, discrete measurement [EPM], EndoPilot automatic, continuous measurement [EPA], n = 40/group).

Pulpal access was prepared using water-cooled diamond-coated instruments (Brasseler, Lemgo, Germany), and the occlusal surface ground flat to allow reproducible length measurement. Root canal orifices were coronally enlarged using size 3 and 2 Gates-Glidden-burs (Brasseler GmbH, Lemgo, Germany). After irrigating the pulp chamber with 2ml of 3% NaOCl solution (25°C, 3 min), canals were initially explored until the apical third of the roots was entered using ISO 06 and 08 K-files (VDW, Munich, Germany).

### Endometric measurement

To assess endometric measurements, an established measurement model was used [4, 18]. Teeth were mounted in a measurement chamber, which was then filled with 0.9% NaCl-solution, with roots being completely covered by the solution. For manual measurements, a size #10 K-file was used (VDW), while automatic measurements were performed using a #10 Mtwo file (VDW), with both files being used according to manufacturer’s instructions. Both endometric devices were calibrated and used according to manufacturer’s instructions.

For manual endometric measurement with Raypex 6 (RPM-group), the second green line on the displayed index was used to indicate a working length reaching the AC, while, similarly, a metric measurement result of 36 was used for manual measurements with EndoPilot (EPM-group). Since the EndoPilot allows continuous automatic measurement during rotating instrumentation, the auto-stop function was used for automatic measurements with EndoPilot (EPA-group). During all measurements, root canals were intermittently irrigated with a total of 2ml 3% NaOCl solution per canal.

If the AC had been reached according to the endometric measurement, files were fixed within the teeth using flowable resin (Tetric EvoFlow^,^ Ivoclar Vivadent, Ellwangen, Germany). Teeth were then embedded in acrylic resin (Technovit 4071, Heraeus Kulzer, Wehrheim, Germany), ground and polished (ATM Saphir 360 E, ATM, Mammelzen, Germany) under microscopic observation (STEMI, Zeiss, Oberkochen, Germany) until the middle of the root canal (the maximum width) was exposed.

### Evaluation

**Fig 1:**
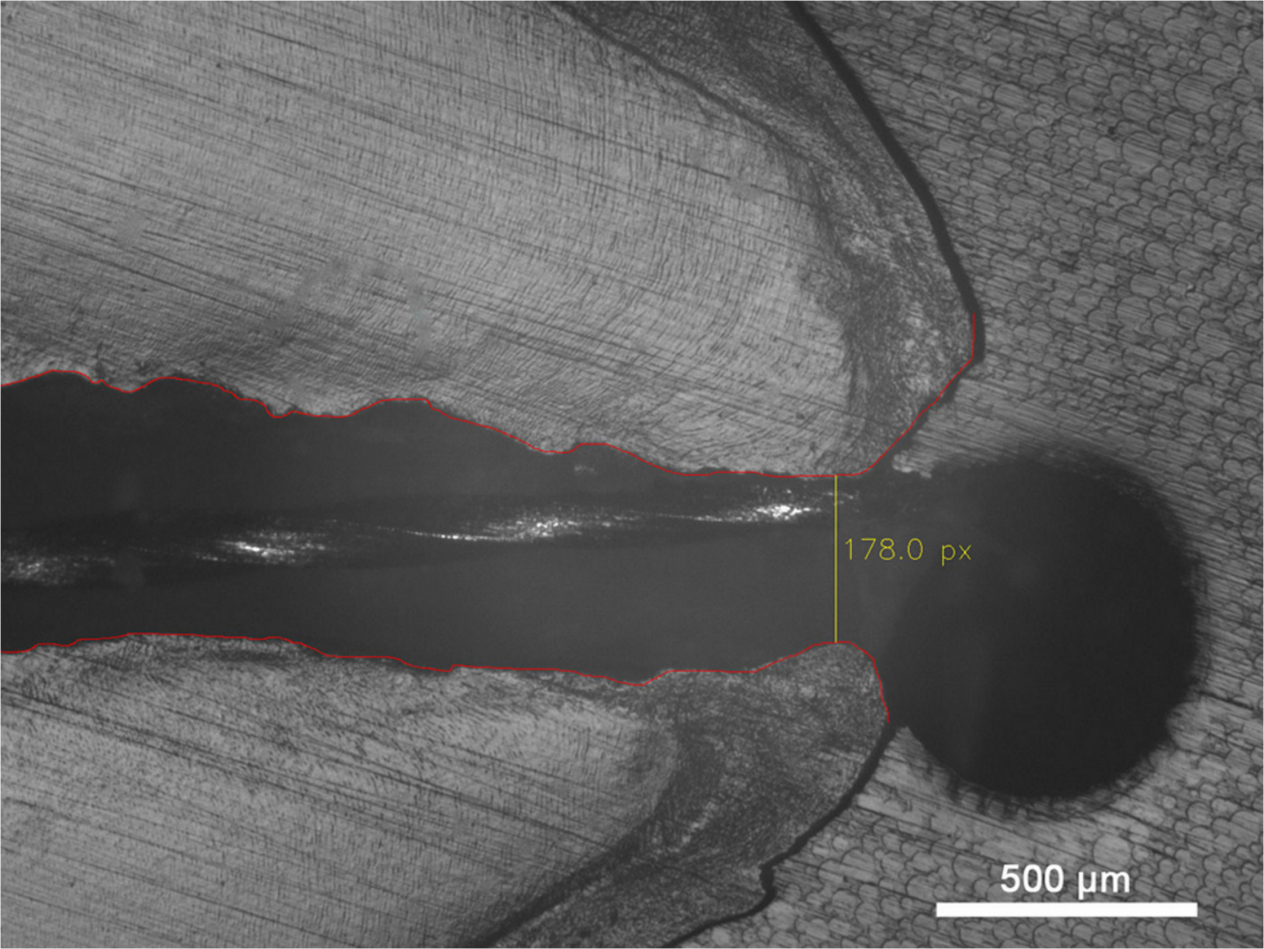
Microscopic, digitalized image of the apex, the periapical region and the endodontic file. The marked outline root canal is shown in red, the computer detected AC and its diameter (in μm) in yellow.

Digital images of cross-sections showing both the tip of the measurement file and the apical area were assessed using a microscope (Axiophot 2, Zeiss, Jena, Germany) interfaced with a digital imaging system (CFW 1312M, Scion, Frederick, USA) and a personal computer. The images were calibrated and scaled using AxioVision (Zeiss, Jena, Germany). The walls of the root canals were digitally marked (Adobe Photoshop CS5.1; Adobe, Munich Germany); the smallest distance between both walls was determined using an experimental software system (Fig 1).

**Fig 2:**
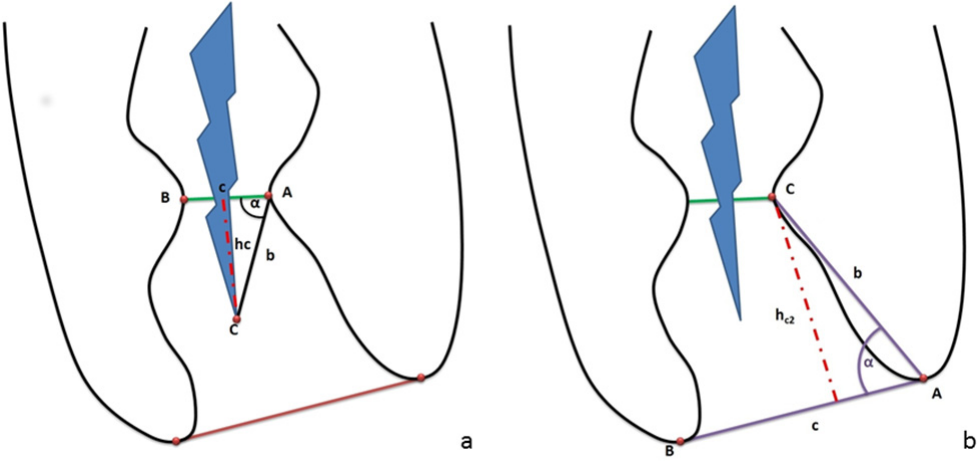
Trigonometric analysis of the distance between the tip of the file and the AC (D_AC,_ a) or the AF (D_AF,_ b), respectively.

The diameter of the apical constriction and the apical foramen were measured. The distance (D) between the tip of the file and AC or the apical foramen (AF) was calculated using trigonometric analysis with the formula (Fig 2).

D=b×sin⁡∝

Using a similar approach, the mean distance between AC and AF was determined (Fig 2). The digital evaluation was independently performed by two dentists, and evaluation of 20 random images was additionally repeated after two weeks to allow calculation of inter- and intra-examiner reliability using intra-class-coefficients (ICC). Subsequently, it was analyzed if the tip of the file was positioned between AC and AF, before AC, or beyond AF, respectively.

Data were statistically contrasted using GraphPad Prism 6.0 (GraphPad Software, La Jolla, USA). To assess the influence of the endodontic measurement system on D, we performed one-way analyses of variance (ANOVA). To compare groups, independent samples t-test as well as Fisher’s Exact Test were used. Statistical significance was assumed if p<0.05.

## Results

The computer-aided measurement was shown to be a consistently reliable method. Both intra- and inter-rater reliability were near perfect, with mean (95% CI) ICC being 0.999 (0.999/1.000, p<0.0001) for detection of both D_AC_ and D_AF_.

Mean (SD) diameters of AC and AF were 0.27 (0.11) mm and 0.64 (0.22) mm, respectively. The mean (SD) distance between AC and AF was 0.36 (0.26) mm. There were no significant differences of these values between groups (p>0.05, ANOVA).

D_AC_ was not significantly different among groups (p>0.05). D_AF_ was significantly smaller for EPA compared with EPM (p<0.01), while RPM showed no difference compared to other groups (Table 1).

**Table 1:** Mean (SD) distance (mm) between the tip of the file and AC or AF, respectively.

	RPM	EPM	EPA
D_AC_	**-0,041**±0,25^a^	**-0,035**±0,27^a^	**-0,064**±0,37^a^
D_AF_	**0,301**±0,22^a,b^	**0,411**±0,28a,	**0,238**±0,35^b^

Different letters along rows indicate significant differences between groups (p<0.05). Negative values indicate positions of the file beyond/apical AC or AF, positive values indicate positions before/coronal these reference points.

In addition, EPA showed the highest frequency of over instrumentation when compared with EPM and RPM (p<0.001), whereas instrumentation aided by RPM resulted in files being positioned between AF and AC in the majority of measurements.

## Discussion

The study compared the accuracy of discrete endometric measurement of Raypex 6 and EndoPilot, and additionally evaluated the accuracy of continuous, automatic measurement of EndoPilot.

The precise determination of the working length is a crucial factor in root canal treatments [21], the endometric measurement provides the most accurate and reliable method [6] compared to tactile and radiographic determination.

A study conducted by Huang showed that endometric measurements rely on electro-physical rather than biological conditions of the surrounding tissues [22]. Thus, *in vitro* simulation of these conditions is suitable to experimentally assess different endometric devices [23, 24]. In addition, it was shown that endometric devices are most likely able to reliably determine the interval between AC and AF [6, 25], whereas the results of this and other studies show that most systems do not reach this aim yet [15].

In this study the analysis of both the apical anatomy and the relative position of the file using a digital system based on image analysis allowed an objective, reliable and precise evaluation of the accuracy of each measurement method. With the described analysis, both AC and AF can be determined without the need of subjective approximations [18]. In addition, the presented method dramatically decreased the detection limit of experimental analyses [26–28], from mm to μm [29].

Our results concerning the diameter of AC and AF and the distance between both are in accordance with previous studies [12, 13].

However, the two-dimensional assessment of a three dimensional system is a limitation of the present study: Since AC and AF are not planar entities, but rather stretch along a certain root canal length and diameter, such assessment may lead to misinterpretation of the accomplished measurements. Furthermore, a three-dimensional detection of this landmark using for instance a micro-CT might offer some advantages [30, 31], but would cause less accurate results than the two dimensional assessments [32, 33].

The results showed high accuracy for both devices, if they were used for manual determination of the working length. However, none of the devices seemed to offer significant advantages when compared to other commercial systems [17]. While less than 5% of the canals had been overprepared when endometric assessment was done manually-discrete, the automatic, continuous measurement led to significantly more overpreparation (24%). This might be due to the apically driven working mode of rotating instruments; a slow motion mode approach in proximity to AC might have some benefits. Further studies should assess other rotating endodontic systems with continuous automatic endometric measurements. In addition, future research may address how the rotating files themselves affect the manual-discrete measurement, and if continuous endometric assessment is more accurate without the auto-stop function.

## Conclusions

This study found high accuracy for RPM and EPM, with no significant differences between the systems, whilst continuous automatic measurements were more often associated with over preparation than manual, discrete measurements. This might be associated with the auto-stop function used and should be further analyzed. The employed image-analysis-based assessment system has high reliability and is suitable for the comparison of endometric measurement devices in vitro.
